# Chronic Oxidative Stress Promotes Molecular Changes Associated with Epithelial Mesenchymal Transition, NRF2, and Breast Cancer Stem Cell Phenotype

**DOI:** 10.3390/antiox8120633

**Published:** 2019-12-11

**Authors:** Ana Čipak Gašparović, Lidija Milković, Nadia Dandachi, Stefanie Stanzer, Iskra Pezdirc, Josip Vrančić, Sanda Šitić, Christoph Suppan, Marija Balic

**Affiliations:** 1 Division of Molecular Medicine, Ruđer Bošković Institute, HR-10000 Zagreb, Croatia; lidija.milkovic@irb.hr; 2 Department of Internal Medicine, Division of Oncology, Medical University, Graz 8036, Austria; nadia.dandachi@medunigraz.at (N.D.); stefanie.stanzer@gmail.com (S.S.); christoph.suppan@klinikum-graz.at (C.S.); 3 Outhospital Emergency Medicine Department of Krapina Zagorje County, HR-49000 Krapina, Croatia; iskra1511@gmail.com; 4 Institute of Cancer Sciences, University of Glasgow, Glasgow G12 8QQ, UK; josip.vrancic@glasgow.ac.uk; 5 Cancer Research UK Beatson Institute, Glasgow G61 1BD, UK; 6 Sestre milosrdnice University Hospital Centre, University Hospital for Tumors, HR-10000 Zagreb, Croatia; sanda.sitic@yahoo.com

**Keywords:** breast cancer stem cells, 4-hydroxy-2-nonenal, extracellular matrix, NRF2

## Abstract

Oxidative stress plays a role in carcinogenesis, but it also contributes to the modulation of tumor cells and microenvironment caused by chemotherapeutics. One of the consequences of oxidative stress is lipid peroxidation, which can, through reactive aldehydes such as 4-hydroxy-2-nonenal (HNE), affect cell signaling pathways. On the other hand, cancer stem cells (CSC) are now recognized as a major factor of malignancy by causing metastasis, relapse, and therapy resistance. Here, we evaluated whether oxidative stress and HNE modulation of the microenvironment can influence CSC growth, modifications of the epithelial to mesenchymal transition (EMT) markers, the antioxidant system, and the frequency of breast cancer stem cells (BCSC). Our results showed that oxidative changes in the microenvironment of BCSC and particularly chronic oxidative stress caused changes in the proliferation and growth of breast cancer cells. In addition, changes associated with EMT, increase in glutathione (GSH) and Nuclear factor erythroid 2-related factor 2 (NRF2) were observed in breast cancer cells grown on HNE pretreated collagen and under chronic oxidative stress. Our results suggest that chronic oxidative stress can be a bidirectional modulator of BCSC fate. Low levels of HNE can increase differentiation markers in BCSC, while higher levels increased GSH and NRF2 as well as certain EMT markers, thereby increasing therapy resistance.

## 1. Introduction

Tumor cell heterogeneity has been a known fact for a long time, but evidence increasingly suggests that heterogeneity of tumors may be associated with a subpopulation of tumor-initiating cells, also called cancer stem cells (CSCs), as a subpopulation driving tumorigenesis and cancer progression [1]. These cells represent only a small proportion of tumor mass, but seem to have the capability of dissemination and may, for still unknown reasons, reactivate from the quiescent state and cause recurrence of the disease [2,3]. The fate of CSC seems to be highly dependent on their niche and state, either activating or quiescent, which may be determined by their microenvironment. This concept of the tumor being dependent on its microenvironment has been postulated early by Stephen Paget [4], and by clinical trials demonstrating that therapeutic interventions with bisphosphonates positively impact the clinical outcome of breast cancer patients, and confirmed the importance of these interactions [5,6]. Today, a wide array of evidence suggests that the network of interactions between the tumor, the microenvironment with the stroma, the extracellular matrix and the inflammatory cells bidirectionally modulate their tumorigenicity [7,8]. Despite recent advances, interactions between CSC and the microenvironment are difficult to study due to a lack of optimal methods for the isolation of CSC and efficient functional assays, as well as due to a variety of proteins, enzymes, and growth/inhibition factors forming the extracellular matrix (ECM) of the tumor and the CSC niche. In vitro sphere formation assays have been shown to be suitable surrogate models to study CSC biology [9,10].

Numerous factors govern cell growth to generate CSC, and epithelial to mesenchymal transition (EMT) is the process that strongly supports and/or generates the CSC phenotype [11]. EMT is a process normally occurring in embryological development, but if awakened latter in the adult organism, it becomes pathological and generates mesenchymal cells with the ability to migrate [11]. This process is reversible, but in the means of cancer, it is highly undesirable, and EMT and back to mesenchymal to epithelial transition (MET) is the process that causes metastasis [12]. EMT is accompanied by changes in many signaling pathways, which result in differential expression of EMT transcription factors such as snail family transcriptional repressor 1 (SNAIL), snail family transcriptional repressor 2 (SLUG), twist family bHLH transcription factor 1 (TWIST1) [13], but also Nanog homeobox (NANOG), POU class 5 homeobox 1 (OCT4), and SRY-box transcription factor 2 (SOX2) [14]. Studies suggest that these transcription factors, especially TWIST1, can translocate to the nucleus upon increased stiffness of ECM, represented by collagen I [15], which indicates that ECM has a role in this process.

Oxidative stress, a state of increased reactive oxygen species (ROS) production, affects all cell systems. It also represents an important factor contributing to the modulation of tumor cell and microenvironment reactions to chemotherapeutics. Increased ROS may lead to numerous consequences, such as genetic instability, one of the major characteristics of cancer, and the modification of lipids by peroxidation [16]. Lipid peroxidation (LPO) with its end-products—reactive aldehydes—have been increasingly recognized as a biomarker of different diseases, particularly cancer, where mitochondrial HNE plays an important role [17]. In addition, these reactive aldehydes, especially 4-hydroxy-2-nonenal (HNE), are involved in different signaling pathways influencing the cells’ fate (e.g., differentiation, proliferation, or apoptosis) [18,19]. One of the signaling pathways affected by HNE is NRF2/KEAP1(Nuclear factor erythroid 2-related factor 2/Kelch-like ECH-associated protein 1) [20]. NRF2 is an antioxidant transcription factor that is bound to KEAP1 in its inactive state. HNE binds to KEAP1 cysteines and thereby releases its inhibition of NRF2. The release of NRF2 causes its translocation to the nucleus and activation of antioxidant genes’ transcription and consequently enabling cells to survive oxidative challenge [20].

The present study aimed to elucidate if oxidative stress and HNE-modified collagen I, as a representative protein of ECM, in combination with HNE-induced chronic stress influence BCSC. The changes in the frequency of BCSC, antioxidative defense system, and transcriptional and protein expression of EMT markers were evaluated. These changes indicated that different surface modifications and chronic stress may bidirectionally modulate BCSC, supporting either differentiation or stress adaptation.

## 2. Materials and Methods

### 2.1. Cell Line and Medium

SUM159 cells (Asterand, Royston, Hertfordshire, UK), estrogen receptor, progesterone receptor, and Her2negative cell line, with the potential of generating stem-like subpopulation were cultured as mammospheres, according to previous publications [1,2]. Briefly, cells were cultured in Mammary Epithelial Basal Medium (MEBM; Lonza, Basel, Switzerland) supplemented with 10 ng/mL basic fibroblast growth factor (bFGF), 20 ng/mL epidermal growth factor (EGF, both from Peprotech, Rocky Hill, Hartford County, CT, USA), 5000 U/mL heparin (Sigma Aldrich, St Louis, MO, USA) and 20 μL/mL B27 supplement (Gibco/Invitrogen, Waltham, MA, USA) at 37 °C in a 5% CO_2_ humidified atmosphere. Mammospheres larger than 40 μm were collected with 40 μm nylon cell strainers (Corning Incorporated-Life sciences, Durham, N.C., USA) and used for experiments.

### 2.2. Collagen Coating

To test cell growth characteristics on an extracellular matrix (ECM), collagen I was used as an ECM representative protein. Collagen I (Sigma Aldrich, St Louis, MO, USA) was dissolved in acetic acid (50 mM, Kemika, Zagreb, Croatia), diluted in redistilled sterile water in a final concentration of 2 mg/mL and used in the native state or modified by 1 or 10 µM HNE (Enzo Life Sciences, Lausen, Switzerland). Depending on the type of analysis, different formats of cell culture dishes were used with the same coating conditions: Native or modified collagen to its final concentration of 5 μg/cm^2^. Thus, coated cell culture dishes were left to dry in a laminar flow cabinet overnight at room temperature (RT) and subsequently sterilized under UV light for 20 min. Dot-blot analysis with HNE-histidine monoclonal antibody was applied to confirm the binding of HNE to collagen I had occurred (Supplementary Figure S1). After confirmation that HNE did bind to histidine residues of collagen, we proceeded with evaluating the influence of collagen on measured parameters. Cells were also seeded on uncoated surfaces, further referred to as polystyrene (PS).

### 2.3. Cell Seeding and HNE Treatment

Mammospheres were dissociated to a single cell suspension by TrypLE (Gibco/Invitrogen Paisley, UK), and 10,000 cells/100 µL were plated in pre-coated or uncoated cell culture dishes and left to adhere for 3 h. Regardless of the cell culture dish format used, the experimental stoichiometry was maintained in all analyses. The formats of the cell culture dishes were as follows: 96-well microplates (cell viability and proliferation; TPP, Techno Plastic Products AG, Trasadingen, Switzerland); 6-well microplates (qRT-PCR, Western blot; Falcon, BD Biosciences, Franklin Lakes, NJ, USA); 8-well glass chamber slide (immunocytochemical analyses of hormone receptors; Nalgen Nunc Int, Naperville, IL, USA). Cells were then treated with different concentrations of HNE once, for a single treatment, or every 2nd day for 10 days, for multiple treatments. For cell viability and cell proliferation assays, these HNE concentrations varied from physiological (1 to 10 µM) to supraphysiological and pathological (25 to 100 µM). Controls of each growth surface were cultured without HNE. Analyses were performed after 48 h for single HNE treatments and 10 days for multiple HNE treatments as described for each analysis below. After the analysis of cell proliferation and cell viability, 10 µM HNE was selected for further analyses of putative breast cancer stem cell phenotypes, EMT marker expression, and immunocytochemical analyses of hormone receptors and antioxidative defense system. Untreated cells of each coating condition served as controls. All the mentioned analyses are described in more detail below.

### 2.4. Cell Viability—MTT Assay

The cell viability was determined by an MTT-based assay, EZ4U, following the manufacturer’s recommendations (Biomedica, Vienna, Austria). Briefly after the treatment, 48 and 10 days after the seeding, cells were incubated with the MTT dye for an hour, and the absorbance was measured on a plate reader at 450 nm with a reference wavelength at 620 nm (Easy-Reader 400 FW; SLT Lab Instruments, GmbH, Salzburg, Austria).

### 2.5. Cell Proliferation—^3^H-thymidine Incorporation Assay (^3^HT)

The assay was based on the incorporation of radioactively labeled thymidine to the replicating DNA. The assay was performed as described previously [3]. Briefly, cells were treated as described in the previous chapter. ^3^H-thymidine (1 µCi/well) was added to each well 24 h or 9 days after HNE treatment(s) and left for 24 h to allow thymidine incorporation into the DNA. Cells were then harvested, and the rate of ^3^H-thymidine incorporation was measured on a Wallac 1904 DSA liquid scintillation counter (Perkin Elmer, Waltham, MA, USA).

### 2.6. Flow Cytometry Analyses of Putative Breast Cancer Stem Cell Phenotypes

For analyses of putative breast cancer stem cell markers, cells were treated as described above. After 10 days, cells were collected from culture dishes with accutase (PAA Laboratories GmbH, Pasching, Austria). Cells were then incubated for 5 min at 37 °C and rinsed twice with phosphate-buffered saline (PBS). Cells forming mammospheres during the experiments were singularized with TrypLE and finally resuspended in MEBM with supplementation for further analyses.

For the Aldefluor assay, cells were washed, counted, and finally resuspended in Aldefluor buffer [21]. To measure aldehyde dehydrogenase (ALDH) activity, the Aldefluor assay (STEMCELL Technologies, Grenoble, France) was performed according to the manufacturer’s instructions and as previously published [21,22]. Briefly, 2 sets of samples with the Aldefluor substrate BODIPY-aminoacetaldehyde (BAAA) were prepared: (a) control: With diethylaminobenzaldehyde (DEAB, the specific inhibitor of ALDH) and (b) sample: Without DEAB. Controls were used for establishing the background fluorescence of these cells and defining the ALDH-positive region on the Fluorescence Channel 1 (FL1*) vs. the SSC dot plot. The absence of DEAB in the sample group converted BAAA to its fluorescent product, BODIPY-aminoacetate (BAA), defining the ALDH-positive population.

For analyses of CD44 and CD24 expression, cells were incubated with horse serum dilute 1:20 in 6% bovine serum albumin (BSA)/PBS for 30 min. Aliquots of antibodies anti-CD44 Allophycocyanin and anti-CD24 Fluorescein isothiocyanate (BD Bioscience, Schwechat, Austria) at a dilution of 1:40 in a final concentration of 0.08 µg/mL and 5 µg/mL, respectively, were then added and the samples were incubated at 4 °C for 30 min. The cells were washed and stored at 4 °C in the dark until the acquisition on the flow cytometer was performed. The protocol was performed as previously published [2,21]. Cells without staining and isotype controls, all from BD Bioscience, were integrated as controls in all experiments.

All samples were assayed on an LSRII flow cytometer (BD Bioscience), and the data were analyzed with the DIVA software version 8.0.1 (BD Bioscience Concorde Business Park 1/E/1/7, Schwechat, Austria).

### 2.7. Immunocytochemical Analyses of Hormone Receptors

For immunocytochemical analyses, cells were treated as described above. After 10 days, cells were fixed in ice-cold methanol for 20 min, dried, and stored until the staining. Cells were subjected to the antigen retrieval using Tris-EDTA solution, pH 9.0, by heating at 85 °C for 10 min to enable correct epitope folding. The monoclonal mouse anti-human estrogen receptor α (M7047, clone 1D5, DAKO, Glostrup, Denmark) and monoclonal mouse anti-human progesterone receptor (M3569, clone PgR636, DAKO, Glostrup, Denmark), both diluted to 1:50 in 1% BSA/PBS, were used. The secondary antibody EnVision (DAKO, Glostrup, Denmark), was used as recommended by the manufacturer. Finally, the reaction was visualized by DAB (3,3-diaminobenzidine tetrahydrochloride in organic solvent). Nuclei were counterstained by hematoxylin. The positive reaction was evaluated and scored by a trained pathologist (S.Š.) in a blinded manner.

### 2.8. Real-Time Quantitative PCR (qRT-PCR) Analyses of EMT Markers

After the cell treatment for 10 days, total RNA was extracted using a TRIzol Reagent (Invitrogen, Carlsbad, CA, USA) in accordance with the recommendation provided by the manufacturer. Nanodrop was used to quantify and asses the assay for purity (ThermoScientific, Waltham, MA, USA). The reverse transcription of one microgram of total RNA was performed using the QuantiTect Reverse Transcription Kit (Qiagen, Hilden, Germany) following the instructions of the manufacturer. LightCycler 480 (Roche) was used to perform qRT-PCR. Reactions were performed in 20 μL of total volume, consisting of 1× SYBR Green I Master Mix (Roche), 20 nanograms of cDNA as well as 25 μM of each primer (final concentration). All qRT-PCR reactions were conducted in duplicate, and afterward, the values of the quantification cycle were averaged. The comparative Ct method was utilized in the calculation of gene expression. Beta-2-microglobulin (B2M) and lactate dehydrogenase A (LDHA) were used as reference genes with the following primer sequences: B2M forward 5′TGCTGTCTCCATGTTTGATGTATCT 3′, B2M reverse 5′ TCTCTGCTCCCCACCTCTAAGT 3′ (NM_004048.3), LDHA forward 5′ TGTAGCAGATTTGGCAGAGAG 3′, LDHA reverse 5′ CATCATCCTTTATTCCGTAAAGAC 3′ (NM_005566.4). Primer sequences for fibronectin (FN), vimentin (VIM), N-cadherin (N CAD), SNAIL, SLUG, and TWIST were previously published [23].

### 2.9. ROS and Antioxidant Measurements

For ROS and antioxidant measurements, cells were treated as described above. On the 10th day of experiments, cells were incubated with 2′,7′–dichlorofluorescin diacetate (DCFDA) to allow the dye to overload the cells. Excess DCFDA was removed after 60 min when the cells were either incubated with medium alone (control) or with 10 µM HNE. ROS were measured with a Cary Eclipse Fluorescence Spectrophotometer (Varian Australia Pty Ltd., Mulgrave, Victoria, Australia) at λ_ex_ 500 nm and λ_em_ 529 nm.

For antioxidant measurements, cells were detached from the surface by TrypLE, and pelleted by centrifugation at the end of the 10-day treatment. Mammospheres were pelleted and dry pellets of all the experimental groups were stored till analyses. Prior to analyses, cells were lysed by 4 freeze/thaw cycles and afterward were centrifuged to remove cellular debris. Protein levels were then determined according to Bradford [24]. The catalase activity was measured according to the method by Goth with some modifications [25,26]. The activity of catalase was expressed as units per milligram of proteins in cell lysate (U mg^–1^).

For the total GSH content, samples were diluted to 0.03 mg/mL and assayed using a modification of the Tietze method [26,27]. Concentrations of total GSH were expressed as µM of GSH per milligram of total protein (nmol mg^–1^).

### 2.10. Western Blot

In order to perform Western blot analyses, cells were treated for 10 days, as described above. After 10 days, the cells were lysed in RIPA buffer (20 mM Tris-HCl (pH 7.5), 150 mM NaCl, 1% Triton X, 0.5% sodium deoxycholate, 0.1% sodium dodecyl sulfate (SDS)) containing protease inhibitors (Roche Diagnostics GmbH, Mannheim, Germany). The protein concentration of the thus obtained supernatant was quantified according to the Bradford method [24] by measuring absorbance at 595 nm using the microplate reader Multiskan EX (Thermo Electron Corporation, Shanghai, China) and interpolating from the standard curve. Protein samples were mixed with Laemmli buffer, boiled for 5 min at 95 °C and 40 µg of total proteins were resolved on the Tris-glycine SDS-PAGE gels (9% or 10%) and transferred to nitrocellulose membranes (Roti^®^-NC, Carl Roth, Karlsruhe, Germany). Membranes were stained with Ponceau S solution (Sigma Aldrich, St. Louis, MI, USA) for evaluation of transfer efficacy and scanned. Following blocking with 5% nonfat milk (Cell Signaling Technology (CST), Danvers, MA, USA) in Tris-buffered saline (TBS; 50 mM Tris-Cl, 150 mM NaCl, pH 7.6) containing 0.1% Tween-20 for 1 h, membranes were incubated with primary antibodies overnight at +4 °C. The primary antibodies used were: Rabbit monoclonal antibodies for NRF2 (CST:#12721), SLUG (CST:#9585), SNAIL (CST:#3879), NANOG (CST:#4903), OCT4 (CST:#2840), GAPDH (CST:#5174); rabbit polyclonal antibody for TWIST (Santa Cruz Biotechnology, sc-15393); mouse monoclonal antibody for Vimentin (Dako, M0725, Glostrup, Denmark). After incubation with horseradish peroxidase-conjugated secondary species-specific antibodies, immunoreactive bands were visualized using the SuperSignal™ West Pico PLUS Chemiluminescent Substrate (Thermo Scientific, Rockford, IL, USA) and Alliance 4.7 (UVITEC, Cambridge, UK). The analysis software Image Studio Lite (LI-COR, Lincoln, NE, USA) was used for quantification of levels of protein expression. Normalization was made with total proteins (Ponceau S staining) and with GAPDH as a loading control. Results are expressed as relative expression according to non-treated mammospheres (PS 0).

### 2.11. Statistical Analysis

All experiments were performed in at least 2 independent experiments with technical quadruplicates. For both single and multiple HNE treatments, inhibitory concentrations of 50% (IC_50_) were calculated using non-linear regression curve fitting log (inhibitor) vs. response and variable slope with a least square (ordinary) fit, using GraphPadPrism 5 software (GraphPad Software, San Diego, CA, USA). Statistical analyses were performed using two-way ANOVA with Tukey’s post hoc test. Values of *p* < 0.05 were considered significant.

## 3. Results

### 3.1. Effects of Single and Multiple Treatments of HNE on SUM159 Cells Growth

**Figure 1:**
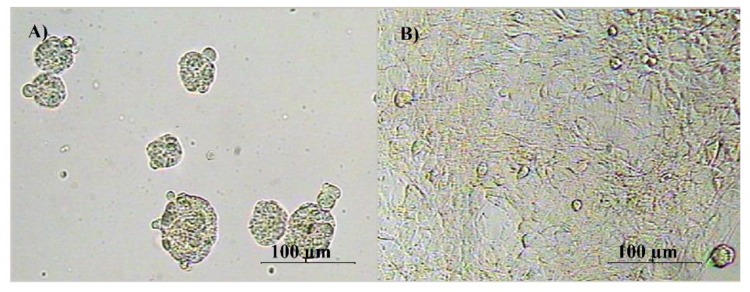
SUM159 cell growth morphology on different growth surfaces. (**A**) SUM159 cells in sphere inducing medium on low attaching growth surface (polystyrene (PS)) and (**B**) SUM159 cells growth in sphere inducing medium on the collagen I coated surface.

We have investigated the effects of single and multiple treatments of HNE as well as the influence of ECM represented by collagen type I, on the SUM159 growth. SUM159 cells grown in mammosphere-inducing conditions formed spheres on PS, in contrast to the adherent spread-like pattern observed on collagen-coated surfaces (Figure 1).

**Figure 2:**
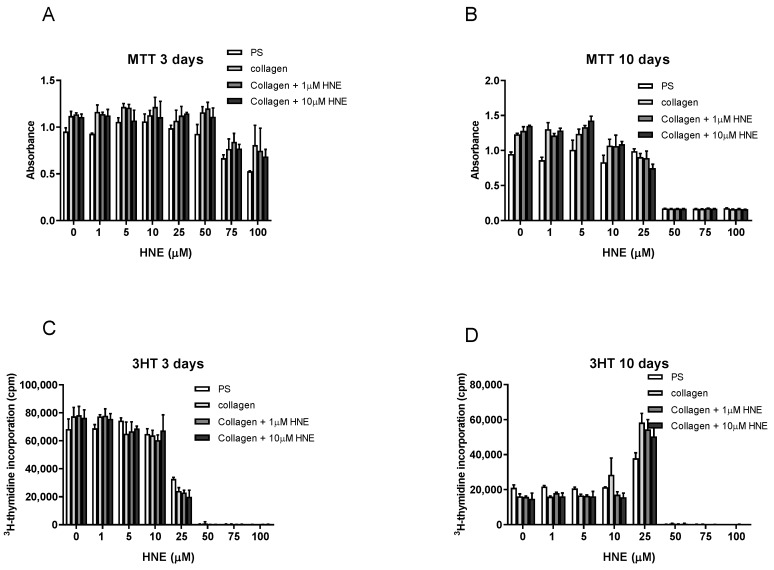
Effects of 4-hydroxy-2-nonenal (HNE) on SUM159 cell growth. SUM159 were exposed to single (**A**,**C**) and multiple HNE treatments (**B**,**D**). Their viability was evaluated by MTT (**A**,**B**), and their proliferation was evaluated by ^3^H-thymidine incorporation assay (**C**,**D**).

The MTT assay showed that SUM159 cell growth in mammosphere inducing conditions on PS had significantly lower viability regardless of HNE concentration used in comparison to coated surfaces and regardless of the time spent in the culture (3 and 10 days) (*p* < 0.05; Figure 2A,B). There was no difference in viability between cells grown on native or HNE-treated collagen when cells were treated with a range of HNE concentrations. The difference was observed in the concentrations causing inhibition, while 100 µM HNE showed inhibition between 50% to 60% after a single treatment, the viability was diminished at 50 µM HNE.

Next, the proliferation of SUM159 cells with the ^3^HT incorporation assay was assessed (Figure 2C,D). While the viability assay distinguished growth on PS and collagen, native, and HNE treated, the proliferation assay did not show any difference in proliferation rates on these surfaces. Inhibition of cell proliferation occurred at similar concentrations of HNE for all growth surfaces (IC_50_ valued presented in Table 1). Multiple HNE treatment did not show differences in proliferation rate on different surfaces. Total growth inhibition was observed at 50 µM HNE and above. Interestingly, 25 µM HNE, which was IC_50_ for single HNE treatment, was stimulating for multiple HNE treatments regardless of the growth surface, reaching more than 200% of the control value. Based on these results, 10 µM HNE was selected, as it did not alter the growth of mammospheres in either single or multiple treatments but did promote cell growth on native and HNE-modified collagen-coated surfaces.

In summary, the basic difference between different growth surfaces was observed by 50% inhibitory concentration (IC_50_) measured by MTT and ^3^H-thymidine assay (Table 1). In single HNE treatment, the IC_50_ could not be determined in MTT assay as there was no total inhibitory concentration applied. In the ^3^HT assay, a slight protective effect was observed for native collagen, and collagen treated with 1 µM HNE (24.05 µM for PS, and 25.54 and 24.83, respectively), while a slight decrease was observed on collagen with 10 µM HNE (23.60 µM). Multiple HNE treatments assayed by MTT sensitized the cells and decreased the IC_50_ to 44.42 µM HNE on PS and to 28.78 µM, 27.74 µM and 26.48 µM for collagen-coated surfaces, native and treated with 1 µM and 10 µM HNE, respectively, while ^3^HT assay showed no difference.

### 3.2. Flow Cytometry Analyses for Putative Breast Cancer Stem Cell Phenotypes

**Figure 3:**
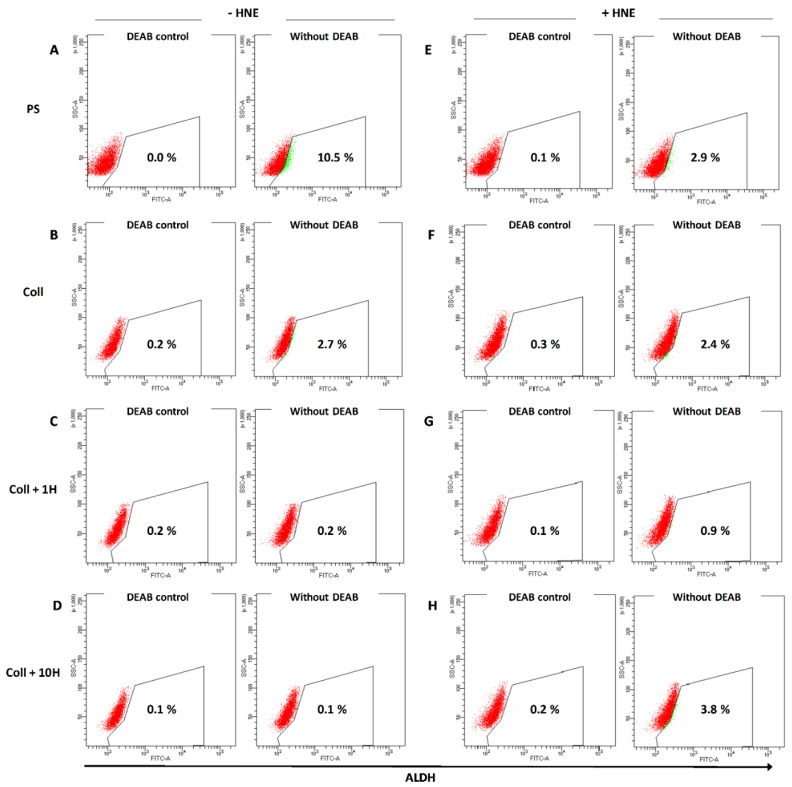
Effects of multiple HNE treatments on the expression of stem cell marker aldehyde dehydrogenase (ALDH) in SUM159 cells. SUM159 cells were cultured for 10 days on different growth surfaces: Polystyrene, PS (**A**), native collagen (**B**), collagen pretreated with 1 µM HNE (**C**), and on collagen pretreated with 10 µM HNE (**D**). Chronic stress was stimulated by the addition of 10 µM HNE every 2 days for 10 days in total on different growth surfaces: Polystyrene, PS (**E**), native collagen (**F**), collagen pretreated with 1 µM HNE (**G**) and on collagen pretreated with 10 µM HNE (**H**). For each panel, both the control and test samples are presented. Control is performed with the addition of diethylaminobenzaldehyde (DEAB), which is an inhibitor of ALDH.

In order to study possible changes in putative cancer stem cell markers due to HNE-pretreated collagen and due to multiple HNE treatments, the expression of CD44, CD24, and ALDH was assessed. The percentage of CD44^+^CD24^–/low^ (results not shown) was concordant with our previous results [21]. There were no significant changes in this phenotype during the treatment. On the other hand, the expression of ALDH-positive cells was different in regard to different growth surfaces and treatment conditions. As presented in Figure 3, untreated cells grown as a mammosphere culture showed the highest proportion of ALDH+ cells (10.5%). Growth on collagen decreased ALDH+ cells to 2.7%, and treatment of collagen with 1µM and 10 µM HNE additionally decreased ALDH+ cells to 0.2% and 0.1%, respectively. Next, HNE treatment was performed in order to assess ALDH activity under stress conditions. There was a decrease observed in the ALDH activity (2.9%) in mammospheres treated with HNE every second day for 10 days. When cells were grown on native collagen, there were small differences between the untreated and HNE-treated cells (2.7% vs. 2.4%). However, in the cells grown on HNE-pretreated collagen and treated with HNE every second day, an increase in ALDH activity was observed compared to the untreated cells on the same growth surface (untreated 0.2% vs. treated 0.9%), with even more pronounced difference of collagen pretreated with 10 µM HNE (0.1% vs. 3.8%). Therefore, our results indicated that HNE-modified collagen, in combination with chronic HNE treatment, caused concentration-dependent responses in ALDH positivity.

### 3.3. Expression of Hormone Receptors

**Figure 4:**
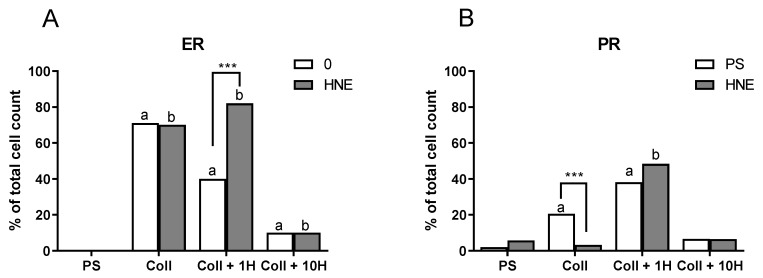
The presence of estrogen (ER) and progesterone (PR) receptors on SUM159 after multiple HNE treatments. After 10 days of treatment with 10 µM HNE every two days, positivity for ER (**A**) and PR (**B**) was evaluated on 1000 cells by the experienced pathologist (S.Š.). All results are expressed as percentages on a 1000 cell count, a—significantly different compared to the control on PS, at least *p* < 0.05, specified in the text; b—significantly different compared to HNE-treated PS at least *p* < 0.05, specified in the text; *** *p* < 0.001 control vs. HNE-treatment on the same growth surface.

As HNE caused concentration-dependent ALDH level changes, we wanted to asses if HNE collagen could induce differentiation. Therefore, we have determined estrogen and progesterone markers (ER and PR) by immunocytochemistry (Figure 4). The ER and PR positivity were validated by an experienced pathologist (S.Š.) by blindfold analysis. Mammospheres were completely negative for ER, while there was some insignificant positivity for PR, regardless of HNE treatment. On the other hand, cells grown on all collagen coatings had ER positivity. The highest ER positivity was observed on native collagen and pretreatment with HNE decreased the number of ER-positive cells in a concentration-dependent manner (40% for 1 µM HNE and 11.4% for 10 µM HNE, respectively). Moreover, treatment with 10 µM HNE did not change already-observed patterns with the exception of collagen pretreated with 1 µM HNE, where the treatment additionally increased the percentage of positive cells (40% vs. 80.2%). PR positivity was similar to ER, very low on PS. Growth on collagen increased PR positivity, with the highest levels on collagen pretreated with 1 µM HNE, and the lowest for 10 µM HNE. HNE treatment showed different trends, which were surface-specific: Increased PR positivity on PS and collagen pretreated with 1 µM HNE, decreased on native collagen, while it did not affect PR positivity on collagen pretreated with 10 µM HNE. These results are in line with ALDH results.

### 3.4. Antioxidants and ROS

**Figure 5:**
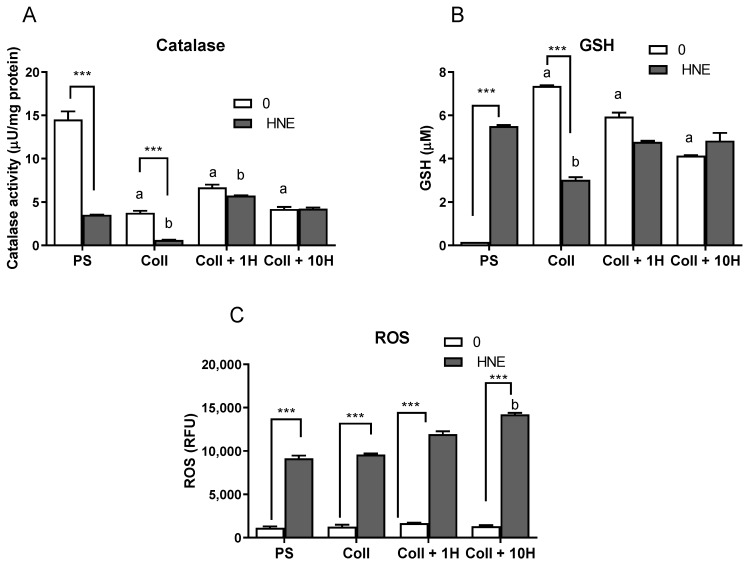
Effects of multiple HNE treatments on the catalase activity (**A**), Glutathione (GSH) levels (**B**), and (**C**) reactive oxygen species (ROS) in SUM159 cells grown on different surfaces. a—significantly different compared to the control on PS, at least *p* < 0.05, specified in the text; b—significantly different compared to HNE-treated PS at least *p* < 0.05, specified in the text; *** *p* < 0.001 control vs. HNE-treatment on the same growth surface.

Further, as cells can adapt to the low level of stress, we have examined parts of the antioxidant defense system, particularly the levels of GSH and the activity of catalase (Figure 5). Catalase activity was the highest in mammospheres, and HNE treatment significantly reduced its activity. In cells grown on collagen, native HNE-pretreated ones had significantly lower catalase activity than in mammospheres (*p* < 0.001). HNE treatment reduced the catalase activity on native collagen while increasing the activity on collagen pretreated with 1µM HNE. Treatment with HNE decreased catalase activity in mammospheres and cells grown on native collagen.

Total GSH levels followed completely different patterns in comparison to catalase, which was expected as HNE is metabolized through the GSH system by binding to GSH [28]. Interestingly, the total GSH levels were the lowest in control mammospheres on PS. Growth on collagen, native, or HNE pretreated, increased GSH levels significantly (*p* < 0.001). HNE treatment decreased total GSH levels on native collagen, and this level was decreased when compared to mammospheres treated with HNE. Interestingly, the two tested antioxidants did not show similar patterns. As the main HNE scavenger GSH was increased with HNE treatment, but also with growth on collagen, native or HNE pretreated, indicating the need for this part of the antioxidant system.

Although both catalase activity and GSH levels varied on different growth surfaces, ROS levels were not changed on different surfaces. HNE addition to cultures significantly increased levels of ROS on all surfaces, with a more pronounced concentration-dependent increase on HNE-pretreated collagen.

### 3.5. EMT Markers

**Figure 6:**
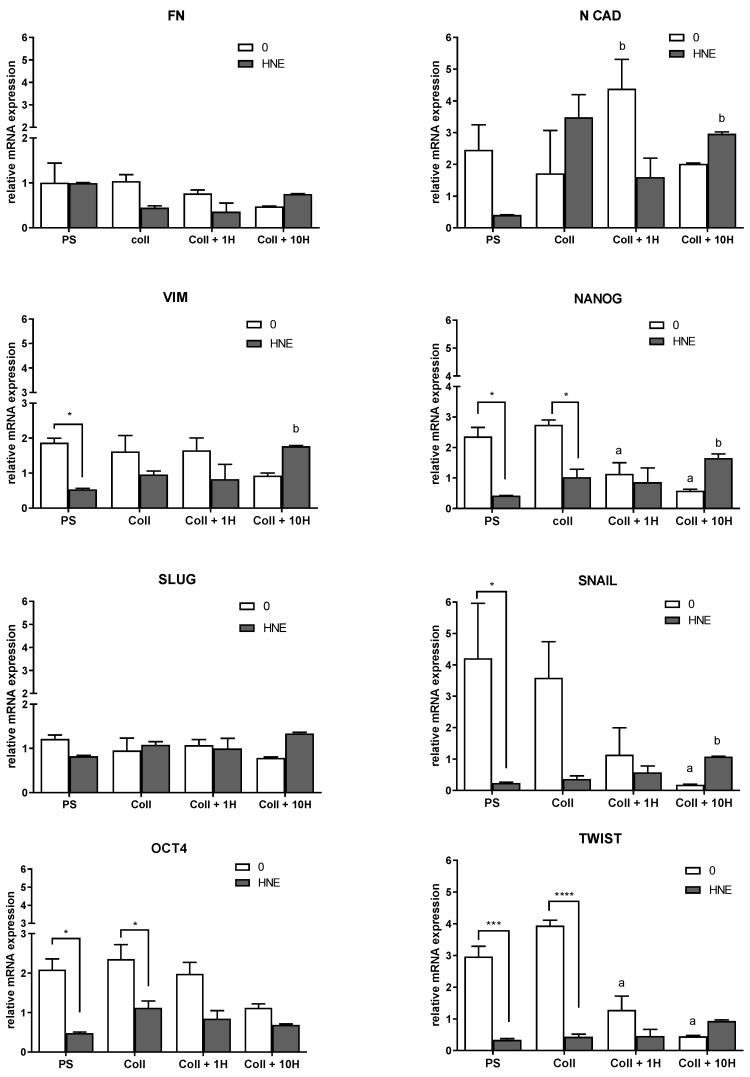
Expression of epithelial to mesenchymal transition (EMT) genes, including N-cadherin (N CAD), vimentin (VIM), fibronectin (FN), NANOG, OCT4, SLUG, SNAIL, and TWIST. The relative mRNA expression was analyzed by qRT-PCR. Bars represent mean +/–SEM of two biological replicates. a—significantly different compared to the control on PS, at least *p* < 0.05, specified in the text; b—significantly different compared to HNE-treated PS at least *p* < 0.05, specified in the text; * *p* < 0.05, *** *p* < 0.001, **** *p* < 0.0001 both control vs. HNE-treatment on the same growth surface.

Changes in the expression of the selected EMT markers were assessed by qPCR, and the results are presented in Figure 6. Among the tested markers, fibronectin, and SLUG did not show any significant changes. The expression of N CAD was significantly increased only in HNE-treated SUM159 cells grown on native collagen (*p* = 0.0096) and collagen pretreated with 10 µM HNE (*p* = 0.0185) when compared to PS. Opposite patterns were observed for vimentin depending on the growth surface conditions and HNE treatment. Repeated HNE-treatment significantly decreased vimentin in mammospheres (*p* = 0.0277). When comparing different growth surfaces/conditions to PS, there was a slight decline within non-treated cells in vimentin levels with increasing HNE concentration, while in HNE-treated cells, surface pretreatments increased vimentin levels especially in cells grown on collagen pretreated with 10 µM HNE (*p* = 0.0416). Similar patterns were observed for NANOG, SNAIL, and TWIST. While multiple HNE-treatments significantly decreased levels of NANOG (*p* = 0.0108), SNAIL (*p* = 0.033), and TWIST (*p* = 0.0004) in mammospheres and NANOG (*p* = 0.0219) and TWIST (*p* < 0.0001) in cells grown on collagen, a slight increase can be observed for all three proteins in cells grown on collagen pretreated with 10 µM HNE. In addition, HNE pretreatment of collagen adversely affected the levels of NANOG, SNAIL, and TWIST in non-treated and HNE-treated cells when growth surfaces were compared to PS. Thus, in cells grown on collagen pretreated with 1 µM HNE, NANOG (*p* = 0.0433) and TWIST (*p* = 0.0027) significantly decreased in non-treated SUM159 cells. The growth on collagen pretreated with 10 µM HNE additionally decreased the levels of NANOG (*p* = 0.006), SNAIL (*p* = 0.0313), and TWIST (*p* = 0.0002) while in HNE-treated cells, the same growth surface increased the levels of these proteins, especially NANOG (*p* = 0.0435) and SNAIL (*p* = 0.0063). Similarly, multiple HNE treatment decreased the expression of OCT4 in cells grown on PS (*p* = 0.011) and on native collagen (*p* = 0.047). Depending on the different growth surface conditions to PS, the levels of OCT4, while revealing similar patterns to NANOG, SNAIL, and TWIST in non-treated cells, differed when cells were exposed to multiple HNE treatments.

As expected, EMT markers were the highest in mammospheres, and HNE treatment either caused no changes or caused a high decrease. Further, collagen and its pretreatment with HNE changed EMT markers, but combinations of HNE treatments and collagen pretreated with 10 µM HNE increased some of the markers to the levels found in mammospheres.

### 3.6. Western Blot

**Figure 7:**
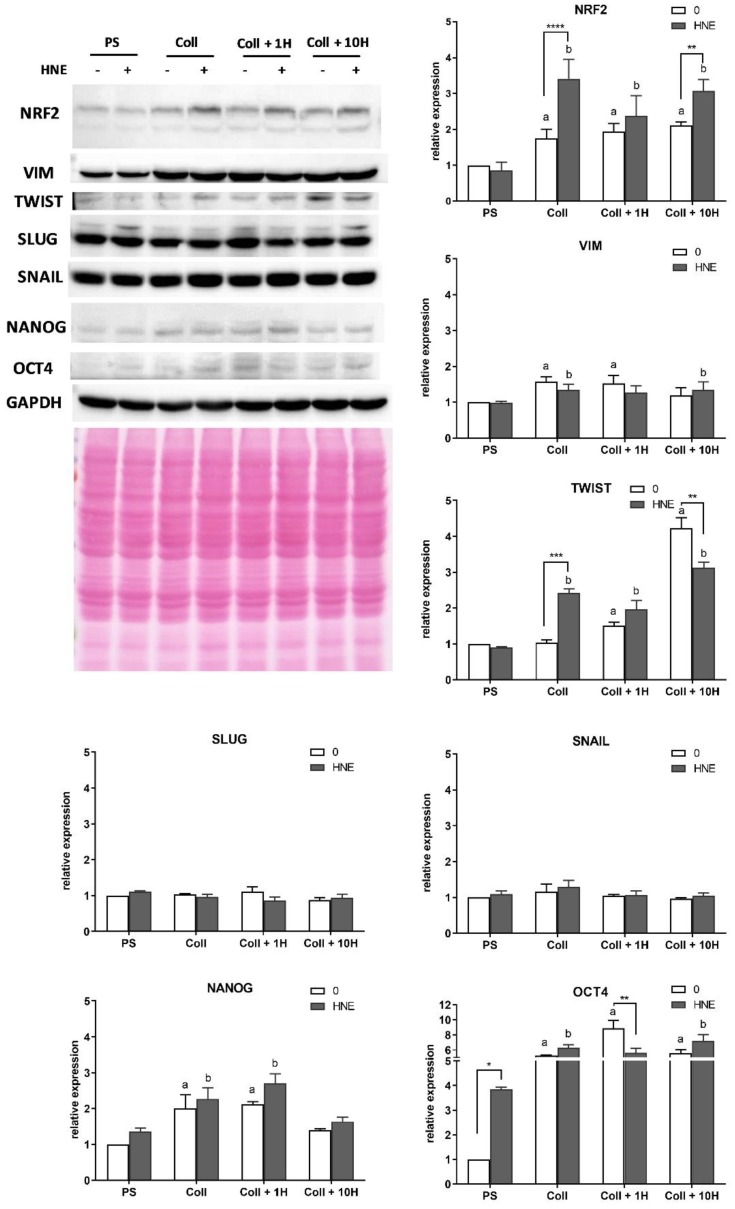
Western blot analyses. Representative blots and relative expression of different proteins: NRF2, VIM, TWIST, SLUG, SNAIL, NANOG, OCT4 are shown. Two-way ANOVA with Tukey’s post hoc test was used to test the differences between groups: a—significantly different compared to the control on PS, at least *p* < 0.05, specified in the text; b—significantly different compared to HNE-treated PS at least *p* < 0.05, specified in the text; * *p* < 0.05, ** *p* < 0.01, *** *p* < 0.001, **** *p* < 0.0001 all control vs. HNE-treatment on the same growth surface.

In order to assess if multiple treatments with HNE caused an increase in antioxidant transcription factor NRF2 levels and to validate mRNA analysis of EMT markers, we performed Western blot analyses of these proteins (Figure 7). For NRF2, it was shown that HNE did not affect its levels when SUM159 cells were grown as mammospheres on PS. In contrast to PS, on collagen, native, or HNE treated, SUM159 cells significantly increased NRF2 levels regardless of HNE treatment (*p* < 0.02 and *p* < 0.0001, for non-treated and HNE-treated, respectively). Multiple HNE treatments additionally increased NRF2 levels in SUM159 cells grown on native collagen and collagen pretreated with 10 µM HNE (*p* < 0.0001 and *p* = 0.0083). In the case of EMT markers, their reaction patterns differed. SLUG and SNAIL did not show any differences regardless of growth surface and HNE treatment. Vimentin was significantly increased in non-treated SUM159 cells grown on native collagen (*p* = 0.0003) and collagen pretreated with 1µM HNE (*p* = 0.001) and in HNE-treated cells grown on native collagen (*p* = 0.0293) and collagen pretreated with 10 µM HNE (*p* = 0.0307) when compared to PS. NANOG showed a similar pattern as vimentin when observing the differences between mammospheres (PS) and different cultivating surfaces. Significant increase of NANOG was observed for both non-treated and HNE-treated cells grown on native collagen (*p* = 0.0055 and *p* = 0.0102) and collagen pretreated with 1 µM HNE (*p* = 0.0029 and *p* = 0.0009), but also without differences between non-treated and HNE-treated cells grown on the same growth surface. HNE seems to be important in regulating the levels of TWIST, regardless of HNE treatment. A significant HNE concentration-dependent increase of TWIST was observed for non-treated cells grown on collagen pretreated with 1 and 10 µM HNE (*p* = 0.0437 and *p* < 0.0001). In the group of multiple HNE treatments, TWIST was increased on all collagen surfaces when compared to PS (*p* < 0.001). Interestingly, multiple HNE treatments increased TWIST levels in cells grown on native collagen (*p* = 0.0003) but decreased them significantly on collagen pretreated with 10 µM HNE (*p* = 0.0014). Among all assayed proteins, OCT4 was the only one significantly increased by multiple HNE treatments on PS (*p* = 0.0128). Additionally, growth on pretreated surfaces increases the levels of OCT4. While in non-treated cells, its levels were increased for all growth surfaces (*p* < 0.0005) in comparison to PS, in HNE-treated cells, OCT4 levels were significantly increased when cells were grown on collagen (*p* = 0.0102) and collagen pretreated with 10 µM HNE (*p* = 0.0013). Surprisingly, HNE treatment significantly decreased OCT4 on collagen pretreated with 1 µM HNE, but both of these levels were higher than on PS.

## 4. Discussion

Cells can, to a certain extent, adapt to numerous stress conditions, and, therefore, the aim of this study was to evaluate whether oxidative stress caused by lipid peroxidation representative end-product HNE has the capability to cause specific molecular changes of tumor cells and impact the frequency of BCSC. Numerous factors may affect tumors, such as oxidative stress, which is a risk factor in tumor initiation and proliferation but can modify tumor microenvironment components, such as proteins and cells, which can further affect tumors. Additionally, a subpopulation of tumor cells, CSC, are increasingly recognized as the main factor of tumor growth and recurrence. Until now, these factors were studied separately. Our findings suggest that HNE modifications of collagen I, in combination with chronic exposure to HNE, may cause changes in the distribution of putative BCSC. Oxidative stress may cause either cell differentiation or, when chronic, an increase of BCSC population and up-regulation of EMT markers.

We have studied the influence of oxidative stress and lipid peroxidation on breast cancer cell line SUM159, modeling both the direct influence of HNE and combinations with modifications of collagen I. The microenvironment of each tumor is unique and the changes in this environment due to inflammation and oxidation processes are complex. Therefore, it is challenging to model these modifications. Oxidative stress is involved in mutagenesis, which is a driving force of (breast) cancer initiation and progression, especially in hereditary breast cancer, where the mere loss of BRCA1 increases ROS [29]. Therefore, it is not surprising that oxidative stress and lipid peroxidation biomarkers are changed in breast cancer patients [30]. HNE is also recognized as a biomarker of oxidative stress, and as such, is involved in (breast) cancer progression [31,32,33]. In accordance with its role are concentrations found in human plasma, where concentrations ranging from 0.1 µM to 1 µM are considered physiological, while 1 µM to 10 µM are considered as ”where pathology begins” [34]. Taken that hereditary mutations in breast cancer, as well as conventional cancer treatment strategies, such as chemo- and radiotherapy, cause increases in ROS, which can, in turn, cause lipid peroxidation and HNE formation, these oxidative processes may affect numerous signaling molecules such as HNE activation of NRF2 transcription factor. In order to study the influence of ECM, we have chosen collagen I, as it can influence some of the EMT markers [15]. We show in our study that collagen may act as a protective agent on SUM159 cell viability, regardless of previous HNE modifications of the collagen. In acute HNE treatment, cell viability was affected at rather higher HNE concentrations (IC_50_ about 100 µM HNE), whereas proliferation was inhibited already at lower HNE concentrations (25 µM), thereby indicating modulation of cell growth and survival. Previously, we observed similar effects of HNE with collagen oxidized by hydroxyl radical instead of HNE [35], indicating that oxidative modifications of collagen I are an important factor when studying cell responses to different stimuli or inhibition factors.

As expected, chronic HNE treatment had a higher impact on cells. Interestingly, proliferation was generally lower in tested cultures than in acute stress, with the exception of 25 µM HNE. This decrease in the proliferation rate after 10 days could be a consequence of increased cell density. In support of this conclusion is the proliferation burst with 25 µM HNE, indicating that these cells adapted and survived the treatment, and, due to the initial decrease in proliferation, now were not spatially limited to grow. Notably, 1 µM HNE, which was considered the physiological concentration, caused differentiation, observed by a decrease in BCSC markers and an increase in hormone receptors, effects that have been described for colon cancer cells and HL-60 cells as well [36,37]. Interestingly, the BCSC marker that we show here, ALDH activity, is the enzyme that can detoxify HNE, particularly ALDH2, which is located in the mitochondrial matrix [17].

Next, we aimed to investigate the influence of chronic stress and HNE modifications of collagen on the expression and protein levels of EMT markers as well as antioxidant parameters measured by catalase activity, GSH levels, and NRF2 antioxidant transcription factor level. Interestingly, although collagen itself, regardless of HNE pretreatment, lowered ALDH, it did not influence EMT markers in the same manner. For example, fibronectin expression was unaffected by different growth surfaces nor by HNE treatment. A similar pattern of expression and protein levels was observed for vimentin, which was increasing with HNE pretreatment concentrations. SLUG was not affected by both mRNA and protein levels. Similarly, SNAIL expression pattern changes were not followed by changes in protein level. Interestingly, expression patterns of TWIST and OCT4 were not followed by protein levels, which were higher in cells on collagen, native, or HNE-pretreated, than on PS. EMT was recognized as an important factor in cancer progression because it represented a conversion between differentiated epithelial cells into migratory mesenchymal cancer cells [38]. The plasticity of CSC enabled them to follow transition traits between EMT and MET, thereby contributing to the metastatic potential of the primary tumor [39]. While many studies link EMT and cancer development and malignancy [40], the influence of oxidative stress/ROS and reactive aldehydes are simply not investigated enough [41]. Numerous factors can stimulate these transitions, and, as shown here, one of them may be chronic oxidative stress.

It was shown previously that EMT might be abolished by the addition of antioxidant curcumin, underscoring the possible role of redox signaling in this process [42]. Therefore, in addition to EMT markers, the levels of GSH, catalase activity, and ROS were measured after HNE treatment and the antioxidant transcription factor NRF2. Interestingly, while catalase activity was the highest in control mammospheres on PS, and decreased by growth on collagen, native or HNE pretreated, GSH levels were significantly increased by both HNE treatment and growth on collagen. It is not surprising that GSH levels were increased by HNE as this is the major scavenger of HNE, and the first step in HNE detoxification [43], while the thioredoxin system is inhibited by HNE and does not contribute to its detoxification [44]. Finally, and in support of GSH increase, were the levels of ROS and NRF2. In all control groups, ROS were at the same level, while the addition of HNE increased ROS, which was additionally increased by HNE pretreated collagen. Following the ROS pattern, growth on collagen increased NRF2 levels, and HNE treatments additionally increased NRF2 on native collagen and collagen pretreated with 10 µM of HNE. HNE is known to activate NRF2 by releasing it from KEAP1 inhibition, and once NRF2 is freed, it translocates to the nucleus [20]. In the nucleus, NRF2 activates transcription of antioxidant genes, among which are glutamate-cysteine ligase, catalytic subunit, and glutamate-cysteine ligase, a modifier subunit, and an enzyme which catalyzes the first step in GSH synthesis [20].

Finally, a recent study indicated that EMT is not the limiting factor for metastasis, but contributes greatly to chemoresistance [45]. Taking all the results into account, our findings indicate that under chronic stress, EMT markers remain elevated and in combination with elevated antioxidant factors such as GSH and NRF2, which can contribute to the maintenance of the BCSC phenotype and therapy resistance.

## 5. Conclusions

Our results suggest that chronic oxidative stress acts as a double-edged sword in supporting the BCSC phenotype. Low levels of HNE can increase differentiation markers in BCSC. In contrast, higher levels and chronic HNE presence increased GSH and NRF2, thereby increasing antioxidative protection. Concurrently, some protein EMT markers are increased, and hormone levels were decreased, thereby supporting the BCSC phenotype and its resistance to oxidative stress. Finally, a better understanding of the role of chronic oxidative stress in the modulation of the breast cancer microenvironment and its impact on breast cancer differentiation may eventually allow for the development of more effective therapeutic strategies.

## Figures and Tables

**Table 1:** Concentrations of HNE being inhibitory for 50% of the treated cells (IC_50_).

Growth Surface	MTT IC_50_ (µM HNE)	^3^HT IC_50_ (µM HNE)
Single HNE treatment		
PS	n.a.	24.05
Collagen I	n.a.	25.54
Collagen I + 1 µM HNE	n.a.	24.83
Collagen I + 10 µM HNE	n.a.	23.60
	Multiple HNE treatments	
PS	44.42	44.59
Collagen I	28.78	45.04
Collagen I + 1 µM HNE	27.74	44.61
Collagen I + 10 µM HNE	26.48	44.64

n.a.—not applicable, concentrations used in the MTT assay did not cause total inhibition. PS—polystyrene.

## Supplementary Materials

The following are available online at https://www.mdpi.com/2076-3921/8/12/633/s1, Figure S1: Dot blot of HNE-collagen I conjugates.

## Abbreviations

HNE: 4-hydroxy-2-nonenal; CSC; Cancer stem cells; BCSC; Breast cancer stem cells; ECM; Extracellular matrix; EMT; Epithelial to mesenchymal transition; MET; Mesenchymal to epithelial transition; ROS; Reactive oxygen species; LPO; Lipid peroxidation; NRF2; Nuclear factor erythroid 2-related factor 2; KEAP1; Kelch-like ECH-associated protein 1; bFGF; Basic fibroblast growth factor; EGF; Epidermal growth factor; PS; Polystyrene; PBS; Phosphate-buffered saline; ALDH; Aldehyde dehydrogenase; MEBM; Mammary epithelial basal medium; RT; Room temperature; EDTA; Ethylenediaminetetraacetic acid; qRT- PCR; Real-time quantitative polymerase chain reaction (PCR); FN; Fibronectin; VIM; Vimentin; N CAD; N-cadherin; B2M; Beta-2-microglobulin; LDHA; Lactate dehydrogenase A; H_2_O_2_; Hydrogen peroxide; GSH; Glutathione; ER; Estrogen receptor; PR; Progesterone receptor
