# Supplementary material for: Chronic Oxidative Stress Promotes Molecular Changes Associated with Epithelial Mesenchymal Transition, NRF2, and Breast Cancer Stem Cell Phenotype
Source: Antioxidants (Basel). 2019 Dec 11;8(12):633. doi: 10.3390/antiox8120633 (PMC6943739; doi:10.3390/antiox8120633)
Supplement: Supplementary file 1 [file antioxidants-08-00633-s001.pdf]

Materials and Methods

Dot-blot

Collagen I solution was prepared and mixed with 4-hydroxy-2-nonenal (HNE) in concentrations 1 μM, 5 μM, 10 μM, 25 μM, 50 μM, 75 μM, and 100 μM according to coating protocol and was left to bind for an hour at room temperature. Following incubation, 100 μL of each mixture was loaded on the nitrocellulose membrane (Bio-Rad Laboratories, Hercules, CA, USA) and washed twice with PBS before the addition of the blocking solution, 2% (*w/v*) nonfat dry milk (Bio-Rad Laboratories, Hercules, CA, USA) in PBS for an hour. The membrane was then incubated with monoclonal mouse anti-HNE-histidine antibody (1:100; clone HNE 1g4, a generous gift of prof. G. Waeg) overnight at room temperature. After incubation with secondary antibody (1:25; EnVision, Dako), immunoreactive bands were visualized using the 3, 3′-diaminobenzidine (DAB; Dako).

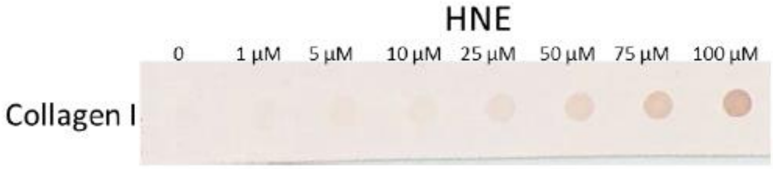

Figure S1. Dot blot of 4-hydroxy-2-nonenal (HNE)-collagen I conjugates.
